# Serum glial fibrillary acidic protein and S100 calcium-binding protein B correlates cerebral vessel reactivity following carotid artery stenting

**DOI:** 10.1038/s41598-021-95867-x

**Published:** 2021-08-11

**Authors:** Xiaofan Yuan, Lei Guo, Jianhong Wang, Duozi Wang, Shu Yang, Fuqiang Guo

**Affiliations:** Neurology Department, Sichuan Academy of Medical Sciences and Sichuan Provincial People’s Hospital, Chengdu, 610072 China

**Keywords:** Diagnostic markers, Prognostic markers, Neurovascular disorders, Stroke

## Abstract

Using detection markers in serum has the advantages of simplicity, repeatability and the capability. This study combined the use of serum glial fibrillary acidic protein (GFAP) and S100B protein (S100B) with imaging tools to confirm the role of serum biomarkers in evaluating the cerebral vessel reactivity after carotid artery stenting (CAS). After CAS, the serum concentrations of GFAP and S100B increased to the peak at 24 h after operation, and then gradually decreased. The mean flow velocity (MFV) (pre-operation, post-operation, 30 days follow-up: 47.65 ± 17.24 cm/s, 62.37 ± 18.25 cm/s, 70.29 ± 16.89 cm/s; P < 0.05) and pulsatility index (PI) (pre-operation, post-operation, 30 days follow-up: 0.78 ± 0.21, 0.98 ± 0.19, 1.02 ± 0.20; P < 0.05) increased significantly in the ipsilateral middle cerebral artery after CAS. At the 30-day follow-up, the cerebrovascular reserve (CVR) (post-operation, 30 days follow-up: 27.47 ± 12.13 cm/s, 31.92 ± 10.94 cm/s; P < 0.05) improved significantly. In patients with different degrees of stenosis, the more severe the stenosis in the carotid artery, the more obvious the improvement of CVR at the 30 days of follow-up (CVR changes: 11.08 ± 7.95 cm/s, Kendall’s tau-b = 0.645, P < 0.001). And the serum concentrations of GFAP (r = − 0.629, P < 0.0001) and S100B (r = − 0.604, P < 0.0001) correlated negatively with CVR at 30 days after CAS. Therefore, we recommend using the biomarkers GFAP and S100B associated with imaging tools such as transcranial Doppler (TCD) and Magnetic resonance imaging (MRI) to evaluate the cerebral vessel reactivity following CAS.

## Introduction

Carotid artery stenting (CAS) is a minimally invasive endovascular intervention that has been advocated for the last 2 decades. Among patients with symptomatic or asymptomatic carotid stenosis, CAS and carotid endarterectomy (CEA) are preventive measures for treating patients with ischemic cerebrovascular disease. The risk of the composite primary outcome of stroke, myocardial infarction, and death does not significantly differ in patients undergoing CAS or CEA^1^. Studies have confirmed that the rate of symptomatic internal carotid artery stenosis is > 50%, the rate of asymptomatic internal carotid artery stenosis is > 80%, and that at least in one patient with high risk factors for CEA treatment, the efficacy of CAS is not inferior to CEA^2^. Three large international multicenter randomized controlled trials also failed to demonstrate that CAS was less effective than CEA^3,4^. CAS is a procedure that patients are more likely to accept because the operation is minimally invasive and requires a shortened hospitalization period^5^.

Astrocytes, which are the main glial cells in the brain tissue, play a role in adjusting neurotransmitters, promoting immune responses, regulating intracranial blood flow, resisting oxidants and so on^6^. Glial fibrillary acidic protein (GFAP) and S100B protein (S100B) are the major components and marker proteins of astrocytes, and both of these proteins are essential to the maintenance of morphological structures and normal functions in astrocytes^7,8^. The increased GFAP and S100B concentrations in cerebrospinal fluid (CSF)^9,10^ and/or blood^11^ reflect the formation of astrocyte filaments in the central nervous system. Rapid rises of GFAP and S100B concentrations suggest the acute injury of brain tissue, and moderate increases suggest the proliferation of astrocytes, the formation of scars, and the delayed ischemic tolerance^12^. Also, moderate increases of GFAP and S100B play an important role in promoting neuronal survival and repairing tissue after brain injury^13^. In summary, researches has demonstrated that GFAP and S100B expressions in CSF and serum are parallel to the size of infarction, the deficit of neurological function, the prognosis of disability after cerebral stroke and the degree of brain injury^14–17^.

In clinical work, transcranial Doppler (TCD) is used to detect intracranial artery stenosis before CAS^18^, monitor the embolus during CAS^19^ and evaluate cerebral hemodynamic changes after CAS^20,21^. Researchers have pointed out that the ischemic brain lesions after CAS could increase the risk of recurrent cerebrovascular events^22^. They recommended the application of diffusion-weighted imaging (DWI) as a surrogate outcome measurement for procedural stroke in carotid revascularization^14^. However, on the one hand, it is not accurate to evaluate the curative effect after CAS according to the velocity of blood flow and imaging of the brain; on the another hand, the two above imaging tools have the disadvantages of poor repeatability, high costs and susceptibility to the operators; additionally, the biochemical detection of body fluids, which can dynamically reflect the changes of brain tissue, can make the results more reliable.

Therefore, this study associated the GFAP and S100B expressions in serum with the TCD and Magnetic resonance imaging (MRI) to evaluate the cerebral vessel reactivity after CAS.

## Materials and methods

### Patients in operation and control groups

From December 2017 to November 2019, patients who were diagnosed with unilateral carotid stenosis and underwent CAS were assigned as the operation group. The inclusion criteria of the operation group are as follows: (1) those with > 50% symptomatic stenosis and the clinical symptoms were consistent with the area of stenosis; (2) patients with ≥ 70% asymptomatic stenosis; and (3) age > 18 years. The exclusion criteria of the operation group are as follows : (1) patients with nervous system related tumor diseases (such as meningioma, gliocytoma, primary or secondary malignant tumor of the brain, etc.); (2) patients with infectious diseases of the nervous system (such as encephalitis, meningitis and myelitis); (3) patients with demyelination and degenerative diseases of the nervous system (such as multiple sclerosis, optic neuromyelitis, Parkinson’s disease and cognitive impairment); and (4) patients with the severe heart, liver, kidney and lung disease who could not tolerate the operation. These patients who were excluded from intra- and extracranial stenosis by Digital Subtraction Angiography (DSA) were allocated to the control group.

### Demographic data

The demographic data of patients include age, sex, diabetes mellitus, hypertension, coronal atherosclerosis heart disease, current smoker, glycated hemoglobin (HbA1c), glycerin trilaurate (TG), total cholesterol (TC), low density lipoprotein (LDL), high density lipoprotein (HDL), uric acid, creatinine and obesity, which were recorded and measured before surgery.

### Operative procedures

Patients in the operation group were given dual antiplatelet therapy (aspirin 100 mg and clopidogrel 75 mg) 7 days before CAS. All of the patients in the operation group received the distal filter protection (Embolic Protection System, Abbott) during CAS, and the stents (Wallstent, Boston Scientific) were successfully implanted by two experienced neurologists. The angiography (Visipaque, 32 g (l)/100 ml, GE Healthcare Ireland, 200 ml per patients) showed that the carotid artery was unobstructed, the stents were well attached to the wall, there was no obvious residual stenosis was found and the anterior blood flow was normal (TICI level 3)^23^. Finally, operative duration was recorded (about 2 h per patients). After the surgery, the patients were prescribed aspirin for a lifetime, in addition to clopidogrel for at least 6 months.

### The standard of carotid artery stenosis

The degree of artery stenosis was independently determined by two neurologists. Disagreement was resolved through discussion, and consensus was reached using a third reviewer. The criteria were assessed in accordance with the standards of the European Carotid Surgery Study Trial (ECST)^24^ and North American Symptomatic Carotid Endarterectomy Trial (NASCET)^25^. The degree of carotid artery stenosis was separated into three grades according to the results of the DSA: Grade 1: 50–70%, Grade 2: 70–90%, and Grade 3: > 90%.

### Evaluation of imaging tools

For patients in the operation group, mean flow velocity (MFV) in the middle cerebral artery (MCA), pulsatility index (PI) which is systolic velocity minus diastolic velocity divided by MFV and cerebrovascular reserve (CVR) on the ipsilateral side were measured before CAS, 24 h and 30 days after CAS. We measured CVR using the breath holding test for a minimum of 15 s after normal inspiration.

MRI was performed before CAS and 24 h after CAS. Also, the scan sequences included the T1-weighted, T2-weighted, fluid attenuated inversion recovery and DWI. The hyperintense, which was presented on the postoperative DWI sequences but not on the preoperative DWI sequences, was considered as new ischemic lesion during CAS.

### Serum sample measurements

A 5-ml of venous blood sample was obtained from the patients in the operative group on the morning of surgery (T1), 24 h after CAS (T2), 72 h after CAS (T3), and 30 days (T4) after CAS. Similarly, a blood sample was collected from the control patients in the morning before DSA (D1) and 24 h after DSA (D2). These blood samples were centrifuged and stored at – 80 °C.

Serum concentrations of GFAP and S100B were measured according to the manufacturer’s instructions. Anti-GFAP (Human GFAP ELISA KIT, ZC-34594, ZCIBIO Technology, shanghai, China) and anti-S100B (Human S100B ELISA KIT, ZC-32056, ZCIBIO Technology, shanghai, China) antibodies were coated in 96 well microporous plates to make solid-phase carriers, and standards or samples were added to the micropores, respectively. The GFAP and S100B were attached to their specific antibody binding on the solid-phase carriers, then after washing, the GFAP and S100B antibodies were added. The absorbance (optical density value) was measured at 450-nm wave length and the serum concentrations were calculated.

### Sample size estimation

Sample size has been calculated by PASS 11. Serum CVR is primary outcome variable in this study. According to previous references, preoperative CVR averaged 20 ± 10 cm/s and postoperative CVR averaged 30 ± 10 cm/s^26^. PASS 11 software calculated that Group sample sizes of 40 in the control group and 80 in the operation group achieve 99% power to detect a difference of − 10.0 between thenull hypothesis that both group means are 20.0 and the alternative hypothesis that the mean of group 2 is 30.0 with estimated group standard deviations of 10.0 and 10.0 and with a significance level (α) of 0.05 using a two-sided two-sample t test^27^. In this study, we actually enrolled 47 patients in the control group and 72 patients in the operation group after excluded the lost follow-up patients.

### Statistical analysis

We performed a statistical analysis using SPSS 23.0 (IBM, USA). Continuous variables were conform to the normal distribution and reported by mean ± standard deviation (χ ± s) values; Continuous variables satisfied the homogeneity of variance, T test and one-way repeated measures analysis of variance (ANOVA) were used for the comparisons between groups and subgroups. The categorical variables were expressed as frequency and percentage, and chi-squared test was used for comparison. We also used the Kendall’s tau-b coefficient analysis and Pearson correlation analysis. A two-tailed P value < 0.05 was considered as statistically significant. The inter-assay coefficients of variation were 6%, 9%, and 10% in GFAP control samples, with mean concentrations of 5.91, 10.73, and 18.92 pg/ml. The 9%, 10%, and 10% in S100B control samples, with mean concentrations of 0.18, 0.31, and 0.57 ng/m, respectively. The mean intra-assay coefficients of variation for duplicate determinations of concentration were 6.7% and 8.6% in serum of GFAP and, respectively.

### Ethics approval

This study was reviewed by the ethics committee of Sichuan Provincial People's Hospital, and all the experimental protocols for involving human data in the study were in accordance with the declaration of Helsinki. All of the patients and representatives/guardian/next of kin of the patients signed the informed consents legally.

## Results

### Demographic data

A total of 72 patients were enrolled in the operation group and 47 patients were enrolled in the control group. There was no statistical difference in demographic data (age, sex, diabetes mellitus, hypertension, coronal atherosclerosis heart disease, current smoker, HbA1c, TG, TC, LDL, HDL, uric acid, creatinine, obesity) between the operation group and the control group (Table 1).

**Table 1:** Demographic data in the patients in the control and operation groups.

Variables	Operation Group (n = 72)	Control Group (n = 47)	T or χ^2^	P
Age (year)	69.34 ± 7.56	64.25 ± 10.83	− 0.359	0.78
Sex (male)	47 (65.28)	27 (57.45)	1.002	0.09
Diabetes	33 (45.83)	10 (21.28)	0.358	0.07
Hypertension	51 (70.83)	21 (70.00)	0.694	0.63
Coronal atherosclerosis heart disease	12 (16.67)	9 (19.15)	0.454	0.32
Current smoker	13 (18.06)	8(17.02)	1.369	0.35
TG(mmol/L)	1.80 ± 0.80	1.50 ± 0.68	0.001	0.10
TC(mmol/L)	4.50 ± 1.81	3.52 ± 1.40	− 1.174	0.49
LDL(mmol/L)	2.03 ± 0.70	1.89 ± 0.78	0.583	0.69
HDL (mmol/L)	2.25 ± 0.43	2.18 ± 0.72	0.557	0.58
HbA1c (%)	5.88 ± 0.40	6.04 ± 0.76	− 2.000	0.11
Uric Acid (μmol/L)	304.23 ± 47.30	325.44 ± 50.68	1.267	0.27
Creatinine (μmol/L)	67.35 ± 12.69	59.39 ± 10.96	2.598	0.22
Obesity	18 (25.00)	9 (19.15)	0.546	0.51

*HbA1c* glycosylated hemoglobin; hypertension, systolic pressure ≥ 140 mmHg and diastolic pressure ≥ 90 mmHg, *obesity* body mass index ≥ 30 kg/m^2^. *TG* glycerin trilaurate, *TC* total cholesterol, *LDL* low density lipoprotein C, *HDL* high-density lipoprotein.

### CAS

**Figure 1:**
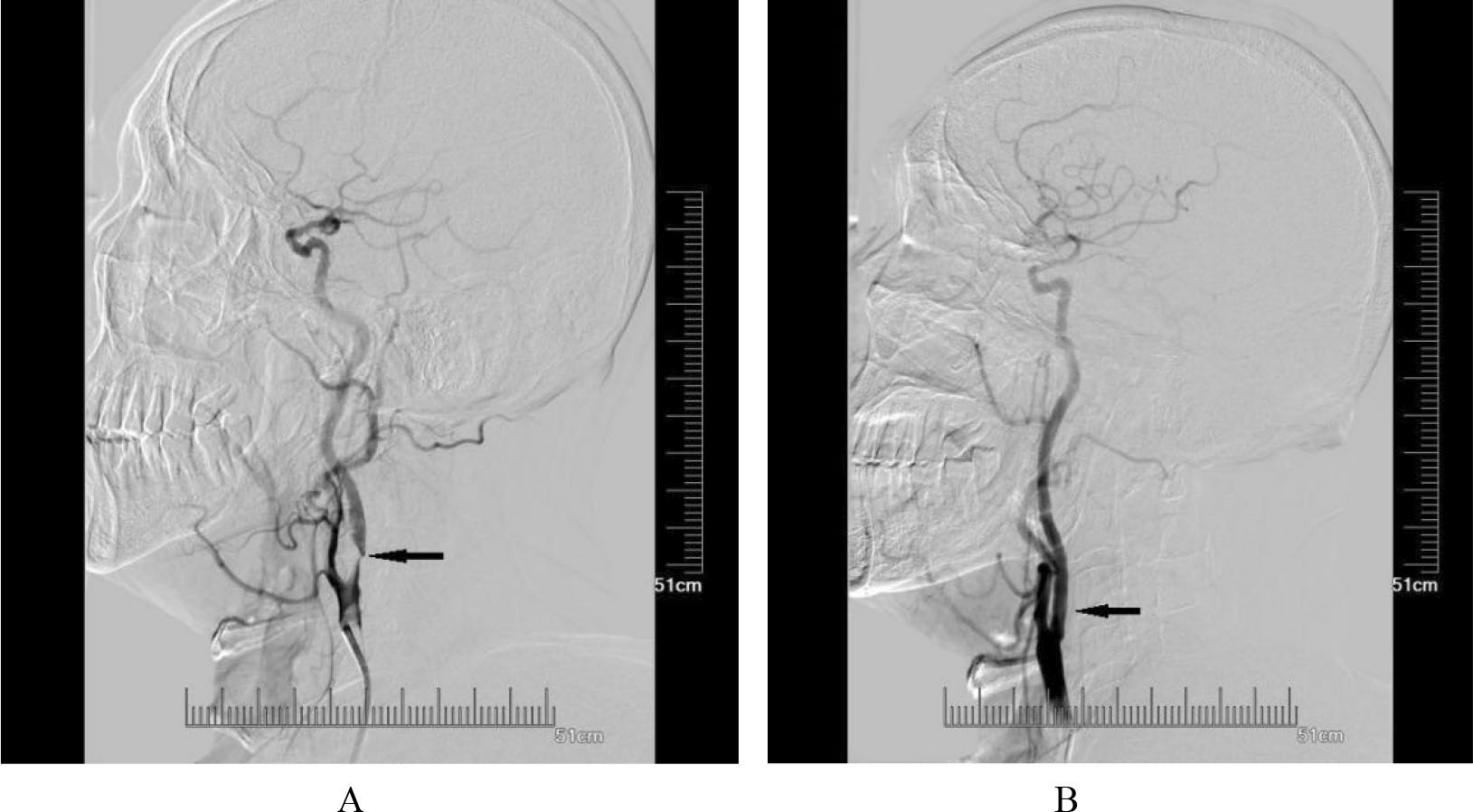
The digital subtraction angiography before and after carotid artery stenting. (**A**) The digital subtraction angiography showed the stenosis in the carotid artery was Grade 3 (> 90%) before carotid artery stenting. (**B**) The stenosis in carotid artery was improved obviously improved and the reperfusion was restored (TICI 3) after carotid artery stenting.

Among the patients in the operation group, 19 patients were diagnosed with carotid artery stenosis—Grade 1, 38 patients were diagnosed with Grade 2 and 15 patients with Grade 3, respectively. The angiography outcomes of carotid artery before and after CAS are shown in Fig. 1.

### TCD

After CAS, the MFV (pre operation, post operation, 30-day follow-up: 47.65 ± 17.24 cm/s, 62.37 ± 18.25 cm/s, 70.29 ± 16.89 cm/s; P = 0.037) and PI (pre operation, post operation, 30-day follow-up: 0.78 ± 0.21, 0.98 ± 0.19, 1.02 ± 0.20; P = 0.018) increased significantly in the ipsilateral MCA. The CVR (post operation, 30-day follow-up: 27.47 ± 12.13 cm/s, 31.92 ± 10.94 cm/s; P = 0.014) improved significantly at the 30-day follow-up. In patients with different degrees of stenosis, the more severe the stenosis in the carotid artery, the more obvious the improvement of CVR at the 30 days of follow-up (CVR changes: 11.08 ± 7.95 cm/s, Kendall’s tau-b = 0.645, P < 0.001). But the CVR (pre-operation, post-operation: 23.39 ± 10.21 cm/s, 27.47 ± 12.13 cm/s; P = 0.179) had no statistical change at 24 h after CAS. The hemodynamic parameters of patients in the operation group evaluated by TCD are shown in Table 2.

**Table 2:** Hemodynamic parameters in the operation patients in the operation group.

TCD values	Pre operation	Post operation	30-day follow-up
MFV(cm/s)	47.65 ± 17.24	62.37 ± 18.25*	70.29 ± 16.89*
CVR(cm/s)	23.39 ± 10.21	27.47 ± 12.13&	31.92 ± 10.94*
PI	0.78 ± 0.21	0.98 ± 0.19*	1.02 ± 0.20*

With the released of carotid artery stenosis and the reconstruction of cerebral blood flow after CAS, the MFV and PI increased significantly in the ipsilateral MCA (*P < 0.05); the CVR improved significantly at the 30-day follow-up (*P < 0.05); CVR showed no statistical difference at 24 h after CAS (^&^P > 0.05).

*TCD* transcranial Doppler, *MFV *mean flow velocity, *CVR* cerebrovascular reserve, *PI* pulsatility index.

### MRI

Although some operative patients exhibited the neurological symptoms before CAS, we performed operation after the stage of acute cerebral infarction for the safety of the operation. So no hyperintense lesion was found in the patients’ DWI images before CAS. However, 29 patients showed the emerging hyperintense in DWI images after CAS. Among the 29 patients with positive DWI, ischemic events occurred in five patients (two patients had TIA, two patients had stroke, and one patient died), and the remaining 24 patients only showed the positive DWI without evidence of neurological deficits. These patients with negative DWI also had no neurological deficits after CAS. There was no evidence of cerebral hyperperfusion syndrome, myocardial infarction and hemorrhage at the 30-day follow-up after CAS.

### Biochemical markers

**Figure 2:**
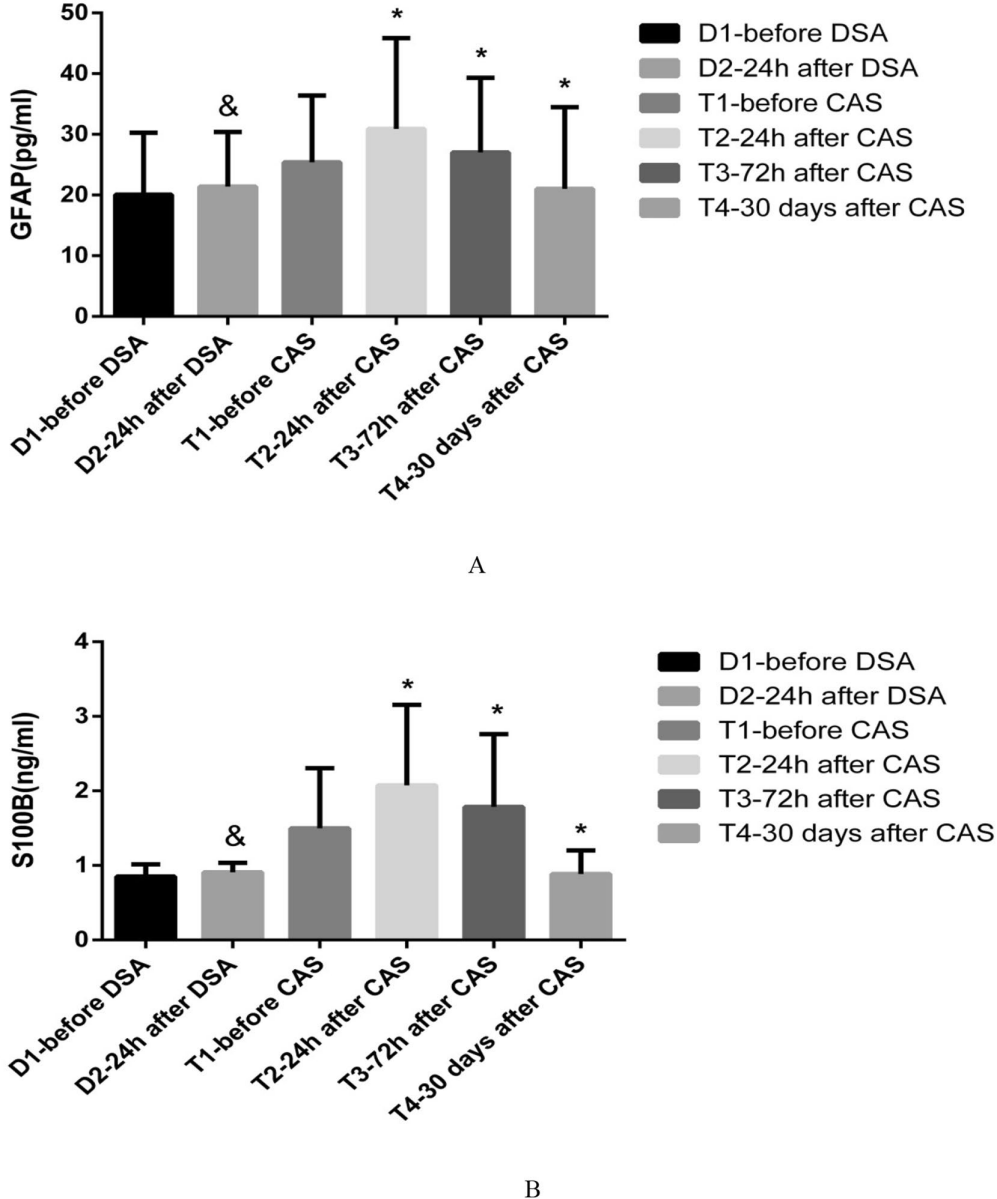
The serum concentrations of GFAP and S100B in patients in the control and operation groups The serum concentrations of GFAP (**A**) and S100B (**B**) did not change significantly after DSA (^&^P > 0.05). After CAS, the serum concentrations of GFAP and S100B increased to the peak at 24 h after operation (T2), and then decreased gradually (T2 > T3 > T4; *P < 0.05).

**Figure 3:**
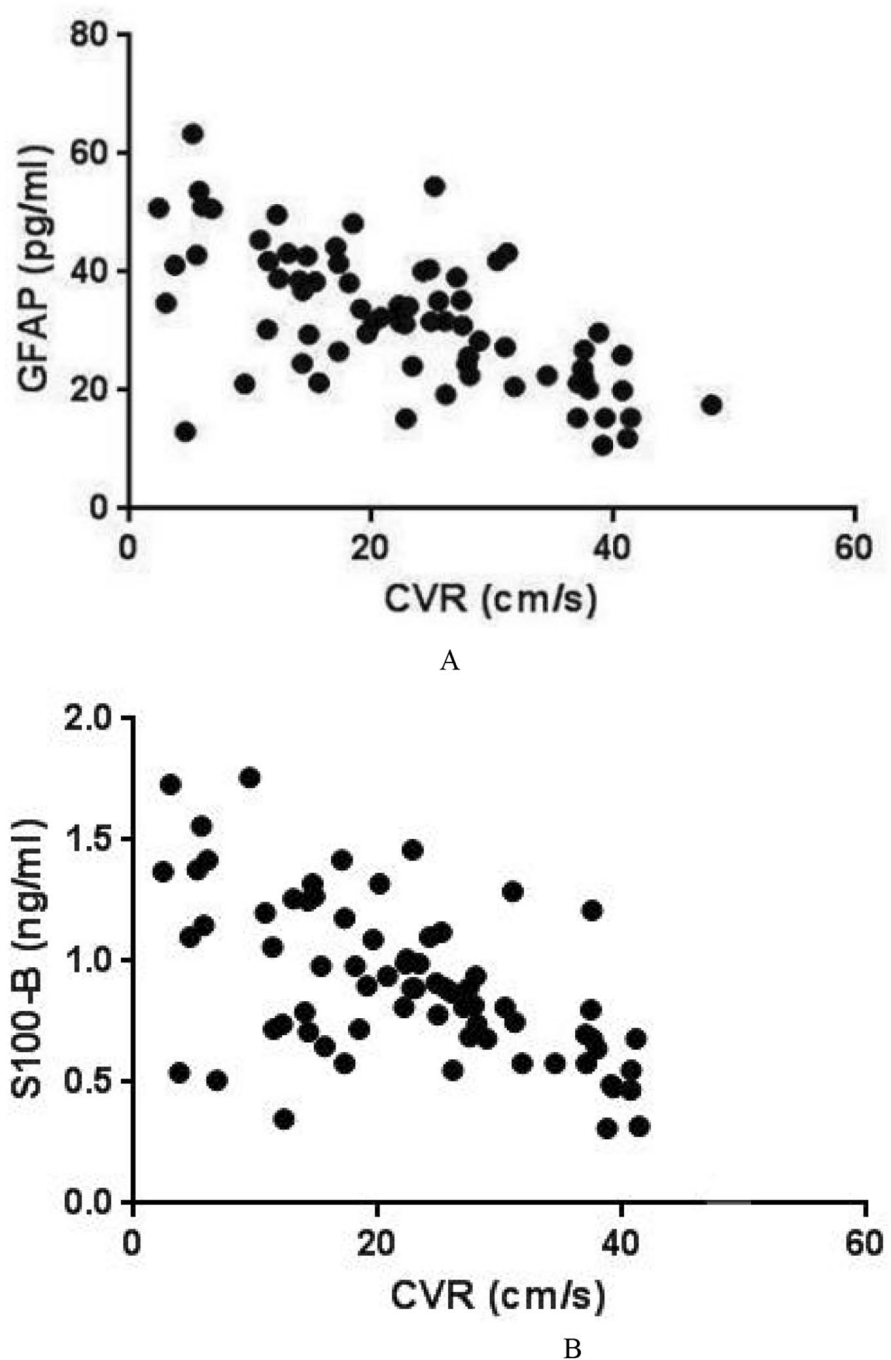
Association between serum GFAP, S100B and CVR at 30 days. Association between serum GFAP (**A**, r = − 0.629, P < 0.0001), S100B (**B**, r = − 0.604, P < 0.0001) and CVR at 30 days after CAS using Pearson’s correlation analysis.

**Figure 4:**
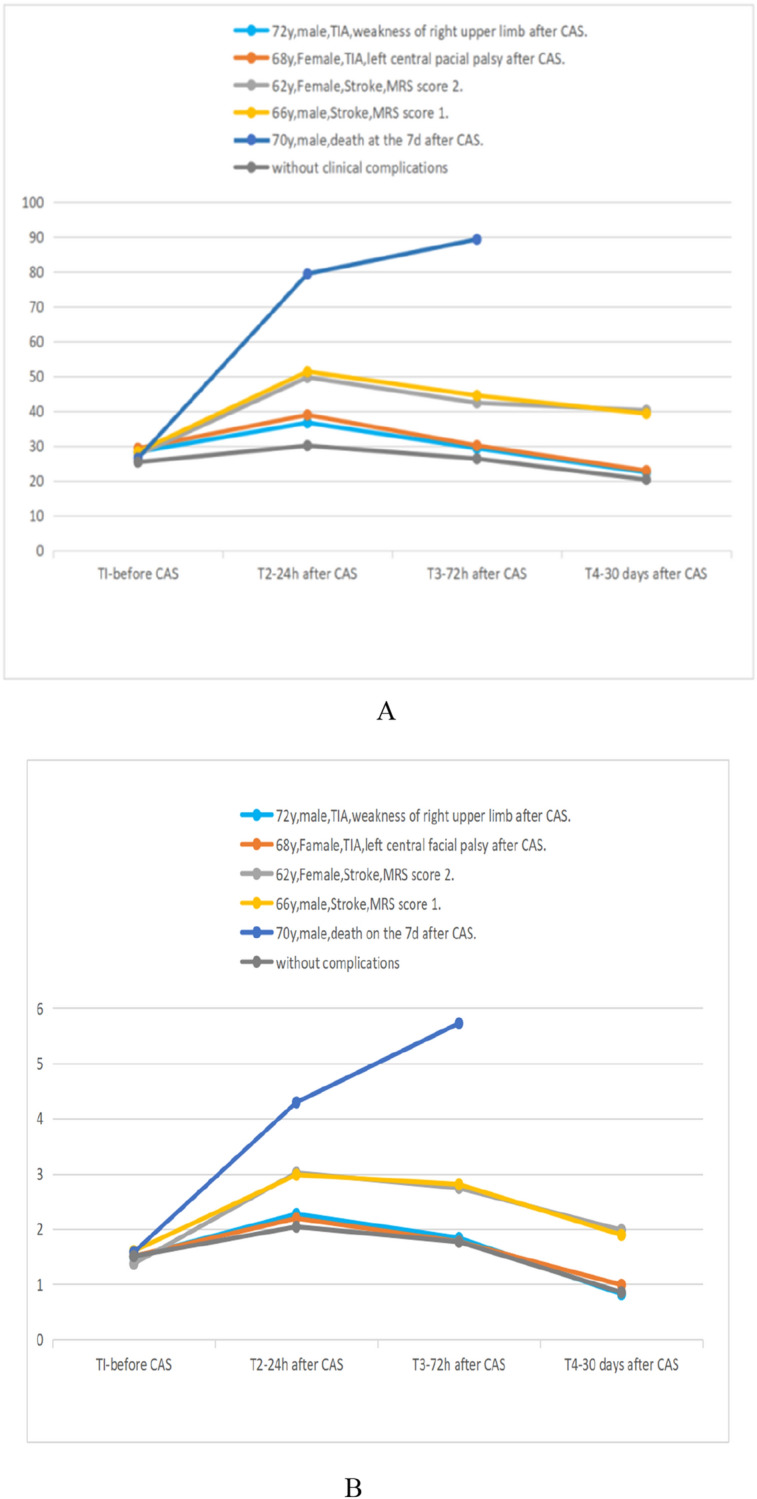
The serum concentrations of GFAP and S100B in five patients with post-operative clinical complications and patients without complications. The serum values of GFAP (**A**) and S100B (**B**) in two patients with TIA increased temporarily at 24 h after operation (T2), and then returned to the basal level at 30 days after operation (T4). For the two patients with stroke and one patient who died, the serum GFAP and S100B increased permanently, and the patient who died maintained a higher level than the patients with stroke at any time points after operation.

(1) There was no significant change in the serum concentrations of GFAP and S100B in the control patients after DSA (P = 0.081). After CAS, the serum concentrations of GFAP and S100B increased to the peak at T2, and then gradually decreased. The lowest values were exhibited at the 30 days after CAS (T2 > T3 > T4; P = 0.014). The serum concentrations of GFAP and S100B in patients in the control and operation groups are shown in Fig. 2. (2) A total of 29 patients showed positive DWI after CAS, and their serum concentrations of GFAP and S100B were higher than patients with negative DWI at T2 (P = 0.019) and T3 (P = 0.030). However, there was no difference in GFAP and S100B at T4 of patients with negative or positive DWI after CAS. The data are shown in the Table 3. (3) The serum concentrations of GFAP (r = − 0.629, P < 0.0001) and S100B (r = − 0.604, P < 0.0001) correlated negatively with CVR at 30 days after CAS using Pearson’s correlation analysis (Fig. 3A,B). (4) Among the five patients with post-operative clinical complications, the serum concentrations of GFAP and S100B in two patients with TIA increased temporarily at T2, and then returned to the basal level at T4. But for the two patients who had a stroke and one patient who died, the serum concentrations of GFAP and S100B increased continuously even at the 30-day follow-up after CAS. The patient who died maintained a higher level than the patient with stroke at any time points during the 1-month follow-up (Fig. 4A,B).

**Table 3:** Serum concentrations of GFAP and S100B in patients with negative/positive DWI after CAS.

Group	MRI	T1	T2	T3	T4
GFAP (pg/ml)	DWI(−)	24.734 ± 11.384	30.039 ± 13.803	26.038 ± 13.294	20.109 ± 12.263
	DWI(+)	25.383 ± 12.723&	32.028 ± 14.927*	28.736 ± 14.829*	21.732 ± 12.728&
S100B (ng/ml)	DWI(−)	1.837 ± 0.937	2.180 ± 1.185	1.623 ± 0.896	0.782 ± 0.289
	DWI(+)	1.802 ± 0.950&	2.478 ± 1.283*	1.732 ± 0.928*	0.872 ± 0.398&

The GFAP and S100B in the serum of patients with positive DWI showed higher concentrations than patients with negative DWI at T2 and T3 (*P < 0.05). However, there was no difference in GFAP and S100B at T4 in patients with negative and positive DWI after CAS (^&^P > 0.05).

*DWI* diffusion-weighted imaging, *CAS* carotid artery stenting.

## Discussion

Carotid artery stenosis is an independent risk factor for cerebral ischemic disease^28^. Patients with impaired cerebrovascular reserve have increased risk of stroke^29^. Stenosis can reduce the blood flow velocity in the local artery and weaken the cerebral vascular reactivity. The disordered hemodynamics caused by stenosis can lead to the occurrence of hypoperfusion cerebral infarction. Furthermore, the micro embolus which is detached from the carotid atherosclerotic plaques can induce the cerebral injury. CAS can not only dilate the stenosis, but also suppress the embolus from vulnerable lesions in the carotid artery even not severe stenosis in the carotid artery.

There are three pivotal compensatory mechanisms for regulating cerebral blood flow^30^. First, the collateral circulation engages in the early stage of chronic hypoperfusion. Second, when the collateral circulation cannot maintain the perfusion, then the CVR begins to participate in the regulation. Third, when the cerebral artery dilation still cannot meet the demands, that is, the CVR fails, the oxygen extraction fraction has to increase and the brain metabolic reserve begins to work. If these above three mechanisms cannot meet the blood and oxygen supply of brain tissue activities, finally the stroke will be occurred. This study utilized the TCD to evaluate the hemodynamics before and after CAS. With the released of carotid artery stenosis, the MFV and PI in the ipsilateral MCA increased significantly after the operation, and the CVR improved significantly at the 30 days after CAS. Furthermore, for patients with different degrees of stenosis, the more severe the stenosis in carotid artery, the more obvious improvement in CVR at the 30-day follow-up. A long-term change requires additional follow-up.

The results in this study showed that the serum concentrations of GFAP and S100B increased temporarily at 24 h after CAS. Meanwhile, 29 patients showed positive DWI after CAS. We speculated that it is the micro-embolism caused by the drop of embolus during the operation^14^. Because astrocytes are sensitive to ischemia and hypoxia in the brain, the micro-embolism could cause the astrocytes to damage and produce excessive GFAP and S100B in the CSF. These proteins as biochemical markers can be released into the peripheral blood through the impaired blood–brain barrier, so the over-expression of GFAP and S100B could be detected in the blood serum^31^. Also, these patients who are demonstrated negative DWI also had increases in both markers in the serum, on the one hand, these results are considered to be the cause of ischemic-reperfusion injury after CAS^32^. On the another hand, the contrast-induced encephalopathy (CIE)^33^ should also be considered. Patients in the CAS and control group were received different doses of contrast agents, an underlying blood–brain barrier disruption, hyperosmolarity, and direct neurotoxicity from the contrast medium may also affect the expression of biomarkers in the serum.

It has been confirmed that the moderate increases of GFAP and S100B in the serum are relevant to the repair after cerebral injury^13^. Therefore, the concentrations of GFAP and S100B at 72 h after CAS were still maintained at a higher level than pre-operation, which suggested that the brain tissue was under repair caused by microembolism and ischemic-reperfusion injury. In addition, it is reported that the reactive gliocytosis has dual effects^7^. When it cannot be solved at the acute injury stage, the reactive gliosis has a negative impact on the injured tissue. If the intervention measures are adjusted correctly within an optimal time frame, new methods for treating cerebral injury may be developed. However, with the release of carotid artery stenosis and the reconstruction of cerebral blood flow after CAS, the injured tissue caused by hypoperfusion was gradually recovered, which was reflected the decreases of both GFAP and S100B at 30 days after CAS. Also, the downtrend of GFAP and S100B after CAS had a negative correlation to the improved hemodynamics, which was verified by TCD. The data in our current study were consistent with the results reported by Wunderlich MT^34^.

The increased concentrations of GFAP and S100B in serum are more significant for patients with symptomatic stenosis (two patients had TIA, two patients had stroke, and one patient died) after CAS than the patients without complications. Furthermore, for these patients who had the neurological deficits (two patients with stroke and one patient who died), the serum concentrations of GFAP and S100B still could not return to the baseline level even at the 30-day follow-up. The patient who died maintained a higher level than the patients with stroke at any time points during the 1-month follow-up, which is different from positive curative patients.

## Conclusion

Our findings has proven that the trend of GFAP and S100B in serum after CAS had a negative correlation to the improved hemodynamics which was verified using TCD. We recommend the biochemical markers (GFAP and S100B) associated with imaging tools (TCD and MRI) for evaluating the cerebral vessel reactivity after CAS.

### Limitations

There are also several limitations in our study. Firstly, the sample size of this study was less than that specified by power calculation. Secondly, we only collected serum samples and monitored the hemodynamics before and after operation, but the long-term data for more than 30 days were lacking. Thirdly, we used the MRI to demonstrate the occurrence of micro-emblism after CAS, but the counts of micro-embolic signals by TCD are lacking.
